# Genome-Wide Detection of Genes Targeted by Non-Ig Somatic Hypermutation in Lymphoma

**DOI:** 10.1371/journal.pone.0040332

**Published:** 2012-07-12

**Authors:** Yanwen Jiang, T. David Soong, Ling Wang, Ari M. Melnick, Olivier Elemento

**Affiliations:** 1 HRH Prince Alwaleed Bin Talal Bin Abdulaziz Alsaud Institute for Computational Biomedicine and Department of Physiology and Biophysics, Weill Cornell Medical College, New York, New York, United States of America; 2 Hematology and Oncology Division, Department of Medicine, Weill Cornell Medical College, New York, New York, United States of America; University of Navarra, Center for Applied Medical Research, Spain

## Abstract

The processes of somatic hypermutation (SHM) and class switch recombination introduced by activation-induced cytosine deaminase (AICDA) at the Immunoglobulin (Ig) loci are key steps for creating a pool of diversified antibodies in germinal center B cells (GCBs). Unfortunately, AICDA can also accidentally introduce mutations at bystander loci, particularly within the 5′ regulatory regions of proto-oncogenes relevant to diffuse large B cell lymphomas (DLBCL). Since current methods for genomewide sequencing such as Exon Capture and RNAseq only target mutations in coding regions, to date non-Ig promoter SHMs have been studied only in a handful genes. We designed a novel approach integrating bioinformatics tools with next generation sequencing technology to identify regulatory loci targeted by SHM genome-wide. We observed increased numbers of SHM associated sequence variant hotspots in lymphoma cells as compared to primary normal germinal center B cells. Many of these SHM hotspots map to genes that have not been reported before as mutated, including *BACH2*, *BTG2*, *CXCR4*, *CIITA*, *EBF1*, *PIM2*, and *TCL1A*, etc., all of which have potential roles in B cell survival, differentiation, and malignant transformation. In addition, using *BCL6* and *BACH2* as examples, we demonstrated that SHM sites identified in these 5′ regulatory regions greatly altered their transcription activities in a reporter assay. Our approach provides a first cost-efficient, genome-wide method to identify regulatory mutations and non-Ig SHM hotspots.

## Introduction

Upon antigen dependent stimulation by T cells, naïve B cells (NBs) are activated, and form germinal centers (GC) within which B-cells (called centroblasts at this stage) undergo massive proliferation and immunoglobulin affinity maturation (AM). AM is mediated by activation induced cytosine deaminase (AICDA), which introduces single and double strand breaks into the immunoglobulin loci during the processes of SHM and class switch recombination (CSR). SHM and CSR can create a pool of diversified antibodies for the antigen by either initiating mutations within the immunoglobulin V region sequences with a frequency that is several orders greater than the basal mutation rate [1] or commencing switches between the different heavy chain constant regions (C_H_) [2]. Periodically, centroblasts enter the light zone of the GC where they interact with dendritic cells and T helper cells to undergo clonal section for cells expressing high affinity antibodies. The high affinity clones survive and differentiate into either plasma or memory B cells, while the low affinity clones undergo apoptosis [3].

The mechanism by which AICDA-induced SHM occurs has been studied extensively [4]. AICDA converts dC to dU, which mimics dT during replication. The mismatch of dU:dG can be processed either by DNA replication to introduce a C-to-T mutation in one of the daughter cells, or by error-prone DNA repair mechanisms, such as base excision repair (BER) or mismatch repair (MMR) to achieve a wider spectrum of mutations at the dU:dG mismatch sites or the flanking nucleotides. Transcriptional activation of the AICDA target loci is required but not sufficient for SHM [5]. Sequence studies of the V region have shown that SHMs are more likely to be transitions (C to T, G to A) than transversions (C to A or G; G to C or T) [6]. In addition, AICDA preferentially targets an RGYW/WRCY (W = A or T, R = A or G, Y = C or T) motif at SHM hotspots on both DNA stands [7], [8], as well as a WA/TW motif [9]. Taken together these results suggest that AICDA-induced SHM is a unique mechanism that B cells adopt to expand antibody repertoire.

Unfortunately, AICDA can also accidentally introduce mutations at bystander loci in B cells undergoing SHM process. SHM hotspots have been observed at non-immunoglobulin loci, particularly within the 5′ regulatory regions of certain proto-oncogenes (*BCL6*, *MYC*, *PAX5*, *PIM1*, *RhoH*, *S1PR_2_*, and *SOCS1*) in diffuse large B cell lymphomas (DLBCL) [10], [11], [12], an aggressive disease comprising approximately 40% newly diagnosed non-Hodgkins lymphomas [13]. This aberrant phenomenon has only been observed in lymphomas arising from B cells that have experienced GC reactions, such as DLBCL, but not in other cancers because dramatic induction of AICDA expression only occurs in these B cells upon antigen stimulation. Due to the limitation of conventional methods, to date this phenomenon has only been studied in a handful of genes. Given that AICDA often remains active in many lymphomas [14], [15], [16], [17], there may be additional genes targeted aberrantly by SHM in DLBCLs. It has been postulated that SHM in these genes may deregulate their expression and contribute to the malignant transformation of activated B cells, however this has only been shown for a handful of genes. For example, SHM-induced mutations in the *BCL6* 5′ regulatory region disrupt BCL6 binding sites and BCL6 self-repression, thus potentially leading to increased BCL6 levels [18]. To identify genes that are targeted by SHM genome-wide, we developed an approach to search for SHM hotspots in the genome integrating next generation sequencing technology with specifically designed computational genomics algorithms. Using this approach we identified new functionally relevant sites of SHM in lymphoma cells. We thus provide a simple and cost-efficient method to screen lymphoma genomes for gene regulatory SHM hotspots and 5′ regulatory mutations, an area that has not been fully explored so far.

**Figure 1:**
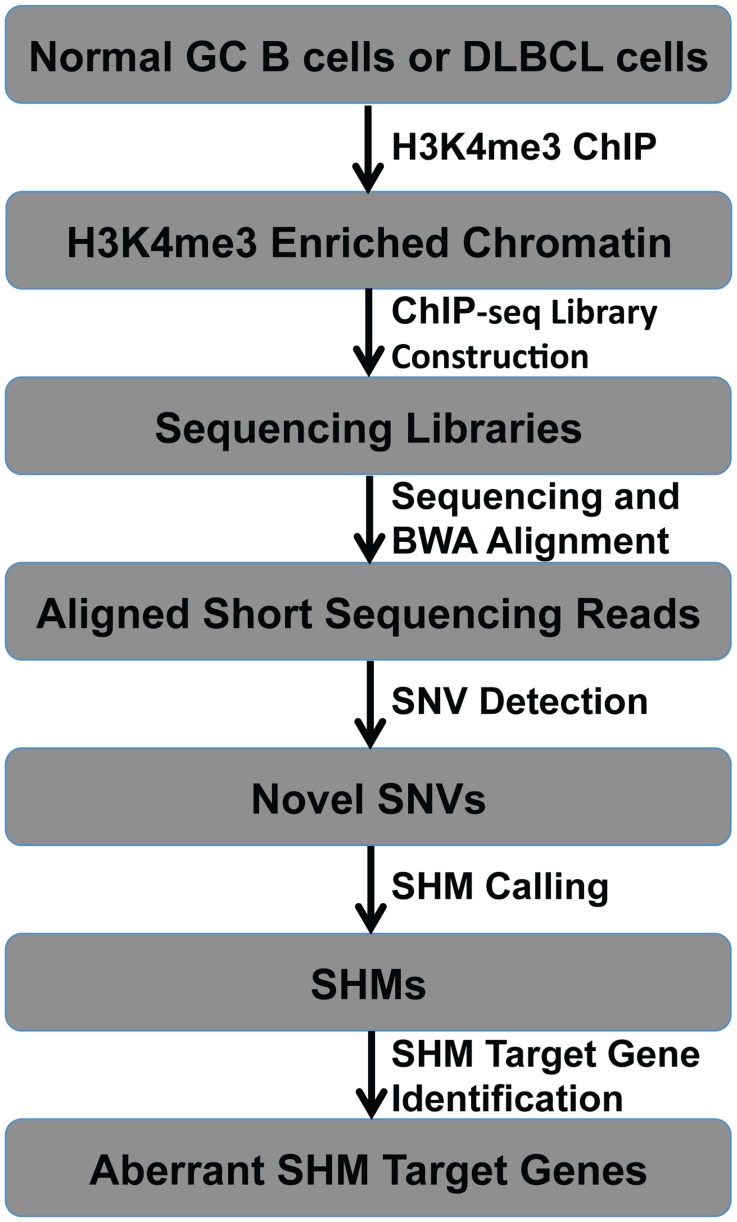
Approach to detect aberrant SHM target genes genome-wide.

## Results

### Using H3K4me3 ChIP-seq Reads for SNV and SHM Calling

SHM typically occurs in the 5′ regulatory region of transcriptionally active genes, especially at locations where chromatin is marked by histone 3 lysine 4 trimethylation (H3K4me3). It also occurs at regions marked with histone 3 lysine 4 dimethylation (H3K4me2), monomethylation (H3K4me1), histone 3 lysine 9 acetylation (H3K9Ac), and histone 3 lysine 27 acetylation (H3K27Ac), all of which are active histone marks that overlap strongly with H3K4me3 at the 5′ regulatory region of transcriptionally active genes [5], [10], [19], [20]. Thus we reasoned that sequencing H3K4me3-associated regions and looking for single nucleotide variants (SNVs) associated with SHM may reveal non-Ig SHM target genes. We anticipated that pulling down H3K4me3-associated genomic regions using an anti-H3K4me3 antibody followed by high-throughput sequencing (ChIP-seq) would provide enough sequence data specifically covering the 5′ regulatory region of actively transcribed genes to detect SNV and SHM within these regions with high accuracy (Fig. 1). To test this hypothesis, we performed H3K4me3 ChIP-seq experiments in two DLBCL cell lines, OCI-Ly1 and OCI-Ly8, and in their normal B cell counterparts, including naïve B cells (NB, IgD+) and germinal center B cells (GCB, CD77+) isolated from human tonsils. After sequencing, short 36-bp (NB, GCB, OCI-Ly1) or 50-bp (OCI-Ly8) reads were aligned to the human genome (hg18) using the BWA short read aligner [21] (Accession Number: GSE34316). We obtained sufficient numbers of uniquely aligned non-clonal H3K4me3 ChIP-seq reads covering a comparable number of 5′ regulatory regions (16,000∼20,000) in all cell types (Table 1). Using the SNV detection approach described in Methods, we found 32,538 high confidence SNVs in NB, 26,258 in GCB, 23,152 in OCI-Ly1, and 14,062 in OCI-Ly8 cells (at least 9× average coverage at these SNVs in each cell type). As expected, a large portion of these high confidence SNVs were within 5′ gene regulatory regions of RefSeq gene (Table 1, 5′ gene regulatory regions are broadly defined as a 4 kb window surrounding transcription start sites, TSS). As also expected, the vast majority of these high confidence SNVs are known polymorphisms in dbSNP release 132 (Table 1). The percentage of SNVs that are known SNPs was similar across all four samples, thus indicating that SNV detection was equally accurate across all four samples (Table 1).

**Table 1:** H3K4me3 ChIP-seq and SNV detection information of NB, GCB, OCI-Ly1, and OCI-Ly8 cells.

	Naïve B Cells	Germinal Center B Cells	OCI-Ly1	OCI-Ly8
Uniquely AlignedNon-clonalReads	25639608	25801467	16181704	3909119
Total SNVs	45567	40087	39740	23663
High Confidence SNVs(% of Total)*	32538 (71.41%)	26258 (65.50%)	23152 (58.26%)	14062 (59.43%)
Percentage of High Confidence SNVs in dbSNP132	74.97%	67.70%	86.93%	80.26%
H3K4me3 EnrichedRefSeq Promoters	20225	16418	19014	18351
High ConfidencePromoter SNVs	11390	6479	15475	8630
Percentage of High Confidence PromoterSNVs in dbSNP132	93.64%	94.35%	91.58%	92.97%
High Confidence Novel Promoter SNVs	724	366	1314	607
Genes Containing High ConfidenceNovel Promoter SNVs#	529	236	841	436

* Filtering by SNV ratio ≥0.33, quality score ≥90, and p≥0.05 for comparison of average quality score comparison between reads that have reference base and reads have putative SNV.

# For the complete list of genes that contain high confidence novel promoter SNVs, please see supporting information.

### Novel 5′ Gene Regulatory SNV Genes in Normal Mature B Cells and DLBCLs

**Figure 2:**
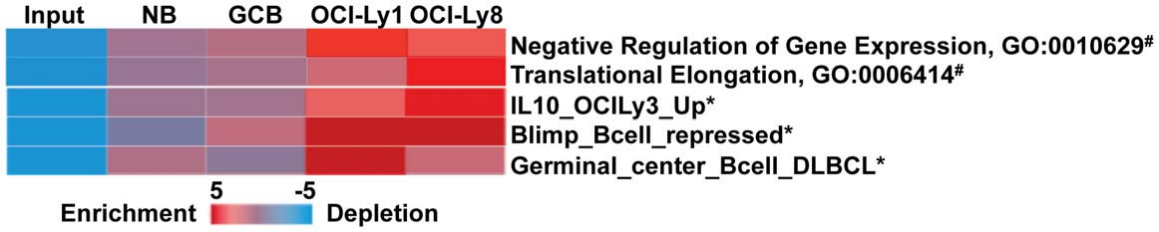
Novel promoter SNVs target important B cell genes in DLBCL. iPAGE analysis using Gene Ontology database (#) [43] or a lymphoid specific gene set database (*) [44] showing strong enrichment of genes that are important for germinal center B cells and DLBCLs contain novel promoter SNVs in OCI-Ly1 and OCI-Ly8 cells.

After SNV detection, we filtered out known polymorphisms from dbSNP 132 and determined the subset of non-dbSNP SNVs in 5′ gene regulatory regions of RefSeq genes. We found 724 such SNVs in NB, 366 in GCB, 1,314 in OCI-Ly1 and 602 in OCI-Ly8 cells (Table 1). We then identified the genes that contain these novel SNVs at their 5′ regulatory regions (Supporting Table S1). We found that the novel 5′ gene regulatory region SNVs obtained from NB were distributed to 529 genes, whereas GCB SNVs were distributed to 236 genes (Table 1), among which 156 genes were common in these two cell populations. Since we isolated these two normal mature B cell populations from the same healthy individual, the difference between the novel SNV spectrums may reflect the difference of the H3K4me3 enrichment locations in these two cell types. In addition, we found 841 genes and 436 genes were targeted by novel 5′ gene regulatory region SNVs in OCI-Ly1 and OCI-Ly8 respectively. Using iPAGE analysis [22], we observed that in DLBCL cells, the genes that harbor these novel SNVs are enriched in gene groups that are related to important biological processes (transcription and translational elongation) as well as important to germinal center B cell and DLBCL biology (Fig. 2). For example, novel 5′ gene regulatory region SNV genes in OCI-Ly1 are enriched in the gene signature of Germinal-center B-cell like DLBCL (Germinal_center_Bcell_DLBCL) identified by Rosenwald et al. (2002) [23]. In addition, novel 5′ regulatory region SNV genes in both OCI-Ly1 and OCI-Ly8 are enriched in genes that are repressed by Blimp-1 (Blimp_Bcell_repressed), a key transcription factor that facilitates the differentiation from GCB to plasma cells by repressing expression of genes important for germinal center B cell function and proliferation [24]. We speculate that some of the 5′ gene regulatory region SNVs identified here may impede the regulation on these genes by Blimp-1, therefore allowing cells to stay in the high proliferating GCB state that may eventually give rise to DLBCL. We also detected an enrichment of OCI-Ly8 SNV genes in the gene expression signature of upregulated genes by IL10/STAT3 signaling pathway in DLBCL [25]. This gene signature includes genes associated with high NF-κB activity, which is known to provide a proliferation advantage to DLBCL cells [26]. Interestingly, although we detected a comparable number of genes that contain novel 5′ gene regulatory region SNVs in normal NB and GCB cells, we could not find any enrichment linked to the functional groups identified above, indicating that 5′ regulatory region of genes key to the normal B cell development are specifically targeted in DLBCL cells (Fig. 2).

### SHM Hotspots in DLBCLs

We then sought to identify the subset of SNVs that have characteristics of SHMs introduced by AICDA, specifically mutations involving the G/C in RGYW/WRCY DNA motifs, or the A/T in WA/TW DNA motifs (where R = A or G, Y = C or T, W = A or T). Because these motifs are reasonably frequent in the human genome, it is not unexpected that a given variant will occur in their context. For this reason, we sought to identify SHM promoter hotspots. We first looked for 5′ gene regulatory regions that have at least three novel SNVs because a Poisson test showed for all cell types that it was unlikely to observe three or more novel SNVs in a 5′ gene regulatory region, given the total number of novel SNVs and the total number of genes marked with H3K4me3 (P<0.005, B–H correction, Table 2; see Methods). We reasoned that not all novel SNV hotspots are SHM hotspots, and that true SHM hotspots should contain a significant number of novel SNVs occurring within an RGYW/WRCY or WA/TW DNA motif context. We therefore identified the subset for 5′ gene regulatory regions where the number of SNVs occurring in RGYW/WRCY and/or WA/TW is at least one standard deviation greater than expected from the local density of these motifs to indicate an enrichment of SHM-like SNVs (see Methods). Only 5′ gene regulatory regions whose SNV status satisfied these criteria were defined as non-Ig SHM hotspots.

**Table 2:** SHM hotspot genes in NB, GCB, OCI-Ly1, and OCI-Ly8 cells.

Cell Type	Gene Name	SNV Number	*p* value (B-H)	SNV in RGYW (Obs)	SNV in RGYW (Exp)	z-score RGYW	SNV in WA (Obs)	SNV in WA (Exp)	z-score WA	Ts/Tv
**NB**	*DUX4*	63(31)	0	6	4	**1.58**	1	3	−1.17	36/27
	*DKFZP686I15217*	6(6)	1.3E−08	1	0	**1.32**	0	1	−0.71	3/3
	*ARL17B*	5(4)	6.5E−07	0	0	−0.6	2	0	**1.96**	2/3
	*TNFRFS14*	3(2)	0.00087	0	0	−0.57	2	0	**4.79**	3/0
	*WASH3P*	3(3)	0.00087	0	0	−0.34	2	0	**5.33**	3/0
	*ARL17A*	3(2)	0.00087	0	0	−0.48	1	0	**1.21**	1/2
**GCB**	*USP17*	35(13)	0	0	2	−1.38	8	4	**2.11**	24/11
	*FRG2C*	5(2)	3.7E−08	1	0	**1.25**	1	1	0.67	0/5
	*DDX11*	3(3)	0.00015	0	0	−0.37	2	0	**4.1**	2/1
	*ARL17B*	3(3)	0.00015	0	0	−0.47	1	0	**1.69**	1/2
	*MIR1324*	3(2)	0.00015	1	0	**1.53**	0	0	−0.7	2/1
	*WASH3P*	3(3)	0.00015	0	0	−0.45	1	0	**1.99**	3/0
**Ly1**	*BCL2*	87(44)	0	10	4	**3.06**	31	11	**6.84**	58/29
	*BCL6*	46(42)	0	5	2	**2.5**	17	6	**5.26**	33/13
	*IGLL5*	30(29)	0	5	2	**3.09**	9	3	**3.93**	17/13
	*S1PR2*	15(15)	0	5	1	**4.87**	2	1	**1.43**	12/3
	*BACH2*	14(11)	0	3	1	**3.08**	1	1	0.03	7/7
	*MYO1E*	13(12)	0	2	1	**1.63**	3	1	**2.41**	9/4
	*BCL7A*	12(2)	0	2	1	**1.24**	1	1	0.04	6/6
	*NCOA3*	11(11)	0	2	1	**1.5**	6	2	**3.38**	6/5
	*BTG2*	9(9)	1E−12	2	1	**2.07**	2	1	**1.9**	5/4
	*RPS17*	6(4)	1.6E−07	0	0	−0.62	2	1	**1.29**	3/3
	*RFTN1*	6(3)	1.6E−07	1	0	**1.12**	3	1	**3.61**	4/2
	*IRF8*	6(5)	1.6E−07	0	0	−0.5	3	1	**2.73**	4/2
	*STARD7*	6(6)	1.6E−07	0	0	−0.69	4	1	**3.36**	2/4
	*FAM86B1*	5(2)	7.3E−06	0	0	−0.53	2	0	**2.73**	1/4
	*ROCK2*	4(2)	0.00022	0	0	−0.42	1	0	**3.16**	4/0
	*RPL23AP53*	4(1)	0.00022	0	0	−0.41	1	0	**1.36**	2/2
	*ZNF596*	4(3)	0.00022	0	0	−0.41	1	0	**1.36**	2/2
	*MEF2B*	4(4)	0.00022	0	0	−0.45	1	0	**1.72**	3/1
	*MRPL45*	3(2)	0.00423	1	0	**2.7**	1	0	**1.21**	1/2
	*CEP63*	3(3)	0.00423	0	0	−0.42	1	0	**1.43**	2/1
	*ARL17B*	3(3)	0.00423	0	0	−0.51	2	0	**3.32**	2/1
	*TCL1A*	3(3)	0.00423	2	0	**3.85**	1	0	0.85	0/3
	*ZFP36L1*	3(3)	0.00423	0	0	−0.41	2	0	**2.68**	1/2
	*ANAPC13*	3(1)	0.00423	0	0	−0.42	1	0	**1.43**	2/1
	*MGC21881*	3(2)	0.00423	0	0	−0.42	1	0	**1.1**	3/0
	*EBF1*	3(0)	0.00423	2	0	**4.71**	0	0	−0.55	1/2
	*MAN2A1*	3(3)	0.00423	0	0	−0.41	1	0	**1.56**	1/2
	*C7orf28B*	3(3)	0.00423	1	0	**2.69**	0	0	−0.58	0/3
Ly8	*BCL2*	38(29)	0	4	2	**1.68**	2	4	−1.19	25/13
	*PARG*	16(5)	0	0	1	−1.04	4	2	**1.4**	9/7
	*PLCXD1*	6(4)	8.6E−09	2	0	**3.54**	0	0	−0.6	4/2
	*CXCR4*	6(5)	8.6E−09	1	0	**1.4**	0	1	−0.72	6/0
	*PIM1*	6(6)	8.6E−09	3	0	**4.53**	0	0	−0.69	5/1
	*WASH1*	4(4)	2.7E−05	1	0	**1.28**	1	1	0.67	3/1
	*WASH3P*	4(4)	2.7E−05	1	0	**1.82**	1	1	0.57	2/2
	*RHOH*	4(4)	2.7E−05	2	0	**3.93**	1	1	0.36	4/0
	*C13orf18*	4(2)	2.7E−05	3	0	**6.44**	0	0	−0.69	2/2
	*RPS17*	3(1)	0.00108	2	0	**4.54**	0	0	−0.77	2/1
	*WASH17P*	3(2)	0.00108	1	0	**1.93**	0	1	−0.85	2/1
	*STAG3L1*	3(3)	0.00108	0	0	−0.34	1	0	**1.28**	1/2
	*BACH2*	3(3)	0.00108	0	0	−0.3	1	0	**1.06**	2/1
	*CIITA*	3(3)	0.00108	2	0	**5.76**	0	0	−0.69	1/2
	*PIM2*	3(3)	0.00108	1	0	**2.98**	0	0	−0.44	3/0

Column 2 lists the genes where non-Ig SHM hotspots were identified. Column 3 shows the novel SNVs identified at the 5′ gene regulatory region of these SHM hotspot genes, the numbers in the parentheses represent the numbers of novel SNVs downstream of TSS. The B-H corrected *p* values estimating the probability of observing the number of mismatches (column 3) by chance are listed in column 4. Column 5 shows the number of novel SNVs observed in the RGYW motifs, whereas column 6 shows the number of novel SNVs expected in the RGYW motifs given the local sequence context. The Z-score for enrichment of novel SNVs within the RGYW motifs of each non-Ig hotspot is listed in column 7. Column 8 shows the number of novel SNVs observed in WA motifs, whereas column 9 shows the number of novel SNVs expected in WA motifs given the local sequence context. The Z-score for enrichment of novel SNVs within the WA motifs of each non-Ig hotspot is listed in column 10. Column 11 shows the numbers of Transition (Ts) and Transversion (Tv) of novel SNVs in the 5′ regulatory region of each non-Ig hotspot gene.

We found 28 such SHM 5′ gene regulatory region hotspots in OCI-Ly1 and 15 SHM hotspots in OCI-Ly8 cells (Table 2, Supporting Table S2). The majority of the novel SNVs at these hotspots occur downstream of TSS (“SNV Number” column, Table 2, numbers in parentheses), a known characteristics of SHM-like SNVs [27]. Several studies have shown enrichment for SHM induced mutations at certain proto-oncogene 5′ regions in DLBCLs, including *BCL2*, *BCL6*, *MYC*, *PIM1*, *RHOH*, and *S1PR2* [10], [11], [12], suggesting a role of non-Ig SHM in lymphomagenesis. Our approach identified all 6 genes as non-Ig SHM targets in our DLBCL cell lines (with similar mutation frequency, Supporting Figure S1) but also discovered several novel SHM target genes of potential relevance to lymphoma. For example, we identified *BACH2*, *BTG2*, *EBF1*, and *TCL1A* as novel aberrant SHM targets in OCI-Ly1 (Table 2). BACH2 is a B-cell specific transcription factor that is required for germinal center formation and blocks plasma cell differentiation by repressing the PRDM1 [28], [29], [30]; BTG2 has anti-proliferative activity [31]; EBF1 is a key lineage determination transcription factor during the transition between pro-B to pre-B cells [32]; and TCL1A, an activator for AKT kinase, is known to be an immunohistochemical marker of adverse outcome of DLBCL [33]. In OCI-Ly8 cells, we identified a different spectrum of non-Ig SHM targets, including PIM2, CXCR4, and CIITA (Table 2). PIM2 is overexpressed in almost 50% of the DLBCLs, and its expression level is associated with the activities of JAK-STAT and NFκB pathways [34]. CXCR4, a chemokine receptor, is a hallmark of germinal center centroblasts, which are the precursors of DLBCL [35]. CIITA is a master regulator of the MHC-II genes whose decreased expression is associated with an adverse outcome in DLBCL patients [36]. Given the importance of these genes in B cells, deregulation of these genes could potentially disrupt normal B-cell differentiation and facilitate malignant transformation. We postulate that the overlapping but overall distinct aberrant SHM target genes seen between these two cell lines may be indicative of the molecular heterogeneity and complexity of biological mechanisms driving the DLBCL phenotype.

**Figure 3:**
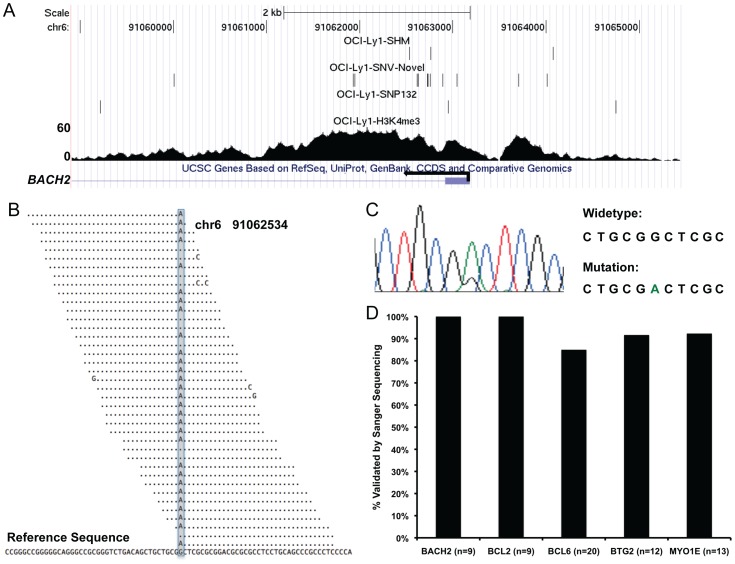
Detection of SHM. (A). A snapshot of UCSC genome browser showing H3K4me3 ChIP-seq reads density at BACH2 promoter. The top three tracks represent SHMs, novel SNVs, and known SNVs (SNP132) detected in OCI-Ly1 by applying SHMseeqer to OCI-Ly1 H3K4me3 ChIP-seq short reads. (B). OCI-Ly1 H3K4me3 ChIP-seq short reads spanning chr6 position 91062534 (*BACH2* intron 1) where a SHM was detected (shaded). (C). Sanger sequencing trace showing detection of both G (wild-type) and A (mutation) at chr6 position 91062534 in OCI-Ly1. (D). Overall validation rates of selected SNVs/SHMs within *BACH2*, *BCL2*, *BCL6*, *BTG2*, and *MYO1E* loci. N indicates number of SNVs/SHMs validated by Sanger sequencing in each locus.

To validate our findings and rule out that the SHM patterns were the result of sequencing errors and/or false positive SNV detection, we performed Sanger sequencing on several SNV-enriched loci in OCI-Ly1. For example, we identified a putative novel SNV in OCI-Ly1 at chr6 position 91,062,534 (hg18), which is located within the first intron of *BACH2* (Fig. 3A). We obtained 39 uniquely aligned ChIP-seq reads spanning this position, among which 29 reads had a G to A change at this position (Fig. 3B). Sanger sequencing clearly showed overlapping G and A peaks at this position (Fig. 3C). We validated additional predicted novel SNVs within the *BACH2* promoter, as well as SNVs in other promoters including *BCL2*, *BCL6*, *BTG2*, and *MYO1E* in OCI-Ly1 cells. Overall, greater than 85% of predicted SNVs were validated (Fig. 3D), demonstrating high accuracy of SNV calling by our approach (see Methods).

### Normal Mature B and T Cells do not Display SHM Hotspots

Since we do not have proper germ-line control DNA for these DLBCL cell lines, we sought to rule out the possibility that the novel SNVs/SHMs are in fact inherited SNPs that would form SHM-like hotspots. Using the same procedure as above for SHM hotspot detection, only six SHM hotspots were found in either NB or GCB cells (Table 2, Supporting Table S2). Although we cannot completely rule out the possibility that the novel SHM hotspots seen in DLBCLs were also present in normal germinal center B cells at a very low level that was beyond the detection confidence of our approach, given the large increase of SHM promoter hotspots in DLBCL cell lines as compared to that in normal GCB cells, our data is consistent with the hypothesis that DLBCL cells have – or have had at some point - altered AICDA activity that aberrantly and more broadly introduces SHM into the B cell genome. Interestingly, we did not observe a correlation between *AICDA* expression level and SHM hotspots numbers. When compared to NB, GCB had 6-fold higher *AICDA* expression measure by RT-qPCR (Supporting Figure S2). OCI-Ly1 had 60% higher *AICDA* expression, whereas OCI-Ly8 had more than 95% reduction of *AICDA* expression as compared to NB (Supporting Figure S2). Although DLBCL cell lines had less *AICDA* expression than GCB cells even though they had more off-target SHM hotspots, it is possible that other deregulated mechanisms alter the genomewide distribution of AICDA binding in DLBCL cells, thus introducing non-Ig SHM. Alternatively, given these cell lines were established from malignant B cells that have already gone through the germinal-center reaction when SHM occurs, these off-target SHMs may have occurred during germinal-center reaction before *AICDA* expression was subsequently down-regulated in these cell lines. As an additional control, we applied our analysis to H3K4me3 ChIP-seq data from CD4+ T cells, obtained from a published study [19]. Upon application of our SNV calling and SHM hotspot detection procedure, we found no SHM hotspots, confirming that aberrant SHM is a malignant germinal center B cell-specific phenomenon.

### SHM Deregulates Target Gene Promoter Activity

**Figure 4:**
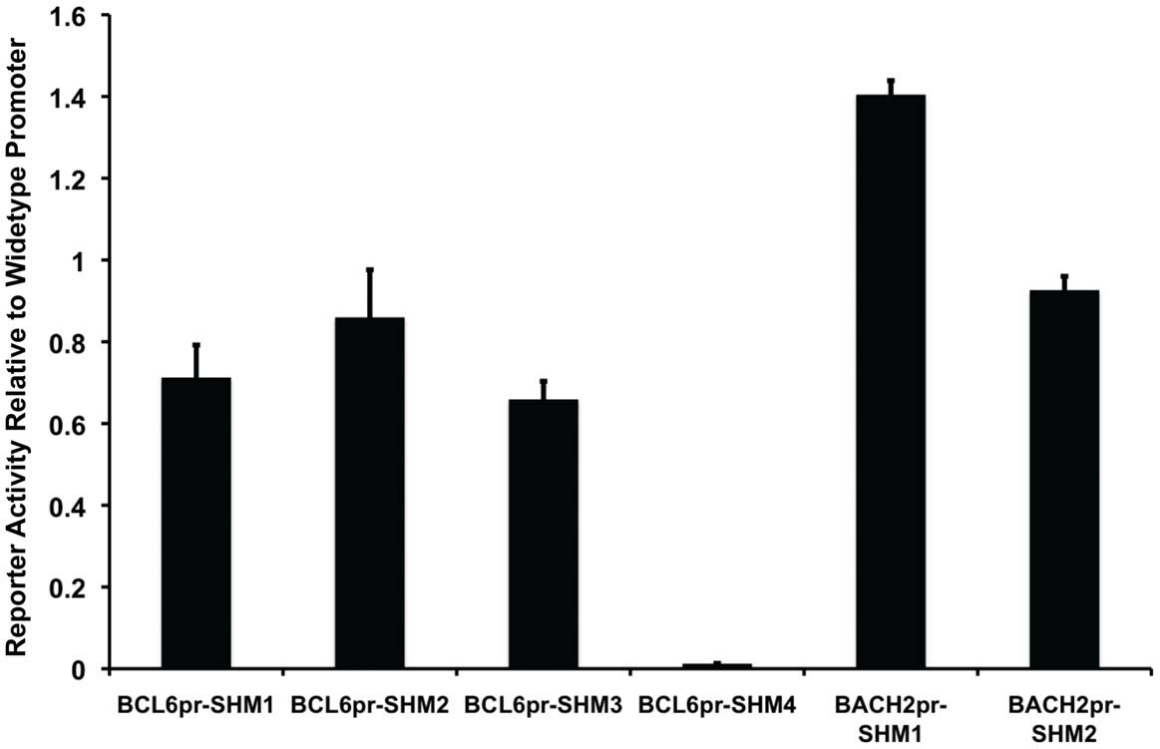
Aberrant SHMs affect promoter activity. The effects of selected SHMs on *BCL6* or *BACH2* promoter activities were tested by dual luciferase assay in OCI-Ly1. Reporter activity of promoter bearing individual SHM was normalized to reporter activity of the wild-type promoter. Error bars indicate standard errors of three independent experiments.

Previous studies identified mutations that disrupt BCL6 binding sites at its own promoter, and these mutations can deregulate a *BCL6* negative autoregulatory loop by interrupting BCL6 binding to these sites [18]. To examine whether SHM discovered by our approach can also deregulate target gene promoter activity, we performed promoter reporter assays on selected SHMs in OCI-Ly1 cells (Fig. 4). We isolated wild-type *BCL6* and *BACH2* promoters from BAC clones, and then performed site mutagenesis at base positions where SHMs have been identified. The activities of the wild-type and mutated promoter were tested in a dual-luciferase reporter assay. Among the four *BCL6* SHMs tested, three reduced the promoter activity by at least 10 to 30% whereas one SHM (BCL6pr-SHM4) completely abolished the *BCL6* promoter activity (Fig. 4). In addition, we tested two SHMs in *BACH2* promoter, one of which significantly increased *BACH2* promoter reporter activity by as much as 40% (Fig. 4), confirming that these non-Ig SHMs can affect target gene promoter activity. Interestingly, we observed great reduction of *BCL6* and *BACH2* expression in OCI-Ly1 cells as compared to their normal counterpart GCB cells (Supporting Figure S3), further suggesting that non-Ig SHMs can affect the expression level of their target genes. However, it should be noted that the expression of these genes reflects not only the mutation status of these promoters but also other ongoing regulatory effects on them in these cells.

## Discussion

SHM at the immunoglobulin locus is an essential process for B cells to generate a large repertoire of high-affinity antibodies. Unfortunately, non-Ig SHM at bystander 5′ gene regions, such as in *BCL6* and in other proto-oncogenes may contribute to the deregulation of expression of these genes and to the malignant phenotype of DLBCLs, which is a unique feature of DLBCLs. Here, we report an integrative approach for the genome-wide identification of SHM and aberrant SHM targets. Using this approach, we confirmed that DLBCL cells have increased 5′ gene regulatory region SHM at non-immunoglobulin loci. Furthermore, we discovered novel SHM targets in DLBCL cell lines. Mutations in these targets may further explain the underlying mechanism of DLBCL tumorigenesis.

Several reasons prompted us to use H3K4me3 ChIP-seq reads as a source of DNA sequences for SNV/SHM detection in our approach. First, H3K4me3 is known to be enriched at the 5′ regulatory region of genes that are expressed or poised to be expressed, where SHMs have been known to accumulate [5], [10], [19]. Second, although SHMs have been shown to be enriched within DNA sequences that are marked by other active histone marks, including H3K4me1, H3K4me2, H3K9Ac, and H3K27Ac, the genome-wide distribution of these marks are highly concordant with that of H3K4me3, particularly at 5′ regulatory regions [20]. Third, because the pattern of H3K4me3 enrichment at gene promoter is highly localized, one lane of single-end 36 bp Illumina sequencing could yield sufficient depth of coverage to obtain high confident mutation calls. This allowed us to significantly reduce the sequencing cost for mutation detection. Fourth, H3K4me3 ChIP is robust, and can be easily applied to patient samples that have limited cell numbers [37]. We expect that with the increasing output of current sequencers and bar-coding technology, it will be possible to achieve cost-efficient mutational profiling of promoters on large cohorts of patient samples with this approach. In addition, although H3K4me3 ChIP-seq does not cover 5′ regions of repressed genes, SNV/SHM information can be extracted from these genes by coupling H3K27me3 (a histone mark known to enrich at repressed gene promoters) ChIP-seq with the approach described here. Furthermore, by applying our approach to datasets of other active histone modifications in DLBCLs, we may identify additional non-Ig SHMs. Indeed, when we applied our approach on H3K4me2 ChIP-seq data from OCI-Ly1 cells, we observed additional SHM hotspots in OCI-Ly1 cells (Supporting Table S3). Eventually, with the reduction of the sequencing cost and the development of personalized diagnosis and treatment, it is foreseeable that our approach could be adapted clinically to identify 5′ gene regulatory mutations in lymphoma patients, which would potentially enable the design of more personalized treatments.

Using the methodology described here, we identified putative SHMs in normal germinal center B cells and two DLBCL cell lines. As expected, we observed low non-Ig SHM hotspots in primary naïve B cells and germinal center B cells. Interestingly, we observed a large increase in the numbers of SHMs in DLBCL cell lines. In addition, we discovered a substantial number of SHM targets in DLBCL cells, including novel targets, such as *BACH2*, *BTG2*, *CIITA*, *CXCR4*, *EBF1*, *PIM2*, and *TCL1A*, that have been shown to play important role in B-cell differentiation and proliferation, suggesting that AICDA-induced SHM is mis-targeted to these genes in DLBCL cells. As mentioned earlier, we cannot completely rule out the possibility that these SHM hotspots are also present in normal germinal center B-cells with a very low abundance that is beyond our detection capacity. However, it is unlikely because when we relaxed the requirement for mutation frequency to 10% (instead of 33%), we could not detect additional SHM hotspots in normal GCBs (data not shown). A recent report by Kato et al. suggested that non-Ig AICDA target genes share three important characteristics with the Ig genes including translocations in tumors, repetitive sequences, and the epigenetic modification of H3K4me3 nearby cleavage sites [38]. We did not observe increased non-Ig SHMs at short repetitive sequences (Table 2, Supporting Table S2 and Table S3). However, the approach used in Kato et al. was designed to identify breakpoints that are introduced by AICDA, a process that resembles class-switch recombination, whereas in our study, we are focusing on SHMs that are potentially introduced by AICDA and subsequent error-prone DNA repair. Therefore, although both types of mutations are introduced by AICDA, we hypothesize that they occur at slightly different genomic loci (CSR at short repeats vs. SHM at RGYW and/or WA motifs).

How AICDA is recruited to these bystander promoters is still largely unknown. Recently, Pavri and colleagues showed that Suppressor of Ty 5 homolog (Spt5), a factor that is associated with stalled or paused Pol II, recruits AICDA to the switch regions of immunoglobulin genes for class switch recombination [20], [39]. Perhaps aberrant binding of Spt5 or other factors to the 5′ regulatory regions of non-immunoglobulin genes may recruit AICDA to these bystander targets and produce disease-driving SHMs in these regions. Alternatively, Liu and colleagues have suggested that roughly 25% of the transcribed genes in normal murine germinal center B cells are targeted by AICDA but shielded from substantial accumulation of mutations by high-fidelity repair [40]. It is possible then certain B cells may have defects in DNA repair pathways, especially in the mismatch repair pathway and the base excision pathway [1], [40], elevating the number of the SHMs occurring at non-immunoglobulin genes. Some of these aberrant SHMs may occur at proto-oncogenes, such as *BCL6* and/or tumor suppressors *i.e. BACH2*, giving cells growth advantages and eventually leading to malignant transformation.

In conclusion, our approach of using histone modification H3K4me3 ChIP-seq reads for genome-wide SHM detection is feasible, cost-efficient, and clinically expandable. Moreover, our bioinformatics detection platform (called SHMseeqer) is highly efficient and accurate for SHM detection, and has powerful and integrative bioinformatics tools for downstream SHM annotation and analysis (http://icb.med.cornell.edu/wiki/index.php/Elementolab/SNVseeqer_Tutorial_SHM). The approach provided here will facilitate our understanding of aberrant SHM and its role in DLBCL malignant transformation.

## Materials and Methods

### Ethics Statement

The de-identified human tonsil tissues were obtained with the approval of the Human Research Protections Programs, Division of Research Integrity of the Weill Cornell Medical College (IRB 0805009767) in accordance with the Declaration of Helsinki.

### SHMseeqer, a Bioinformatics Pipeline for SHM Identification

We developed a bioinformatics platform, called SHMseeqer, for genome-wide detection of mutations that have the characteristics of SHM from ChIP-seq reads (Fig. 1).

#### SNV detection

For each nucleotide in RefSeq transcripts, we calculated the number of overlapping reads (denoted as *n*) and determined how many of these reads showed a mismatch compared to the reference transcript sequence (derived from the hg18 genome sequence). The number of reads with a mismatch is denoted as *k*. We also recorded the position of the *n–k* matches and *k* mismatches within the 36 bp or 50 bp-long reads. We then determined the probability of observing *k* mismatches or more by chance, given the location of these mismatches within the reads and the overall error rate observed at each read position. Because most mismatches are expected to be sequencing errors (as opposed to biological variation), the error rate at position *i*, denoted as *p_i_*, is the number of mismatches occurring at position *i* in the entire sequencing experiment divided by the total number of mapped reads. Under these conditions, the probability of observing *k* mismatches by chance is determined by the Poisson-Binomial distribution:
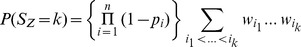
where *w_i_  =  p_i_/(1−p_i_)* for *i = 1…n*. The Poisson-Binomial distribution describes the distribution of sums of Bernouilli variables *S_Z_  =  Z_1_ + ··· + Z_n_*, i.e. where *Z_i_* can take 0 or 1 values, with *p(1) = p_i_*. Modeling error rates at distinct read positions is important because in Illumina sequencing, the number of sequencing errors is often high at the first position in reads and typically increases with the distance from the beginning of reads. Using the Poisson-Binomial ensures that mismatches occurring in read regions with high sequencing error rates get lower weight than mismatches occurring in regions with low error rates. Poisson-binomial p-values, i.e. *P(S_Z_≥k)*, were calculated using the algorithm described in Chen and Liu (1997) [41], P-values were only calculated for transcript positions with sufficient number of reads, i.e n≥4 in this study. To take into account multiple hypothesis testing, p-values were then adjusted using the Benjamini-Hochberg approach and a false discovery rate of 1% was used for SNV calling.

#### SNV annotation

After SNV calling, SHMseeqer annotates the genomic locations of these putative SNVs according to the RefSeq transcripts and filters out known SNPs in dbSNP132. Moreover, SHMseeqer calculates the average sequencing quality score of the reads that contain reference base or those that harbor the SNV base at the putative SNV position, and then uses a t-test to compare the sequencing quality between these two groups of the reads. These measurements provide important metrics to filter out low confidence SNV calls. In this study, we identify those novel SNVs whose average sequencing quality scores ≥90 (based on raw ASCII scores) and the t-test p values ≥0.05 as high confidence SNVs.

#### SHM and SHM hotspots identification

After eliminating known dbSNPs and low confidence novel SNVs, SHMseeqer determines how many novel SNVs are associated with each gene. For each gene, it determines the probability of observing the number of novel SNVs or more, given the total number of novel SNVs and the total number of genes whose 5′ gene regulatory region marked with H3K4me3. This probability is given by the Poisson distribution, 
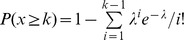
, where *k* is the observed number of novel SNVs, and *λ* is expected number of novel SNVs per 5′ gene regulatory region if those were uniformly randomly distributed in H3K4me3 marked genes. Poisson p-values are then adjusted for multiple testing using the Benjamini-Hochberg correction. This analysis revealed that in the cell types and cell lines we studied, observing 3 or more novel SNVs is highly unlikely to arise by chance (adjusted p<0.005). In genes whose 5′ regulatory region contains 3 or more novel SNVs, SHMseeqer searches for SHMs that occur at dC or dG within WRCY or RGYW motifs, or dA or dT within WA/AT motifs. SHMseeqer then calculates Z-scores to measure the over-representation of SHMs in SNVs within a 4-kb window centered at the transcription start site (TSS) of each RefSeq gene. The Z-scores are calculated by comparing the observed ratio of SHM to the expected ratio, which is determined by permutations to assess the random chance of such mutation occurring in the 4-kb window. Any gene with at least 3 novel SNVs and a Z-score greater than 1 for either RGYW or WA SHMs is designated as preferential hotspot targets of aberrant SHM process. As a final step, SHMseeqer outputs a file that contain the list of the SHM hotspot genes and a file that describes the locations of the SNVs and SHMs within these hotspots.

### Cell Culture

The OCI-Ly1 cells [42] were cultured in medium containing 90% Iscove medium (Cellgro, Manassas, VA), 10% fetal bovine serum (Gemini, Irvine, CA), and 1% penicillin/streptomycin (Invitrogen, Carlsbad, CA). The OCI-Ly8 cells [42] were grown in RPMI with 10% fetal calf serum, 1% penicillin/streptomycin, and N-2-hydroxyethylpiperazine-N′-2-ethanesulfonic acid.

### Tonsilar GC Cell Isolation

Primary cells were purified from normal fresh de-identified human tonsillectomy specimens (IRB 0805009767) by magnetic cell separation based on the expression of phenotypic markers, such as IgD for Naïve B cells, and CD77 for germinal center B cells. Ficoll Histopaque gradient centrifugation was used to isolate tonsilar mononuclear cells. Individual tonsilar B cell populations were collected by antibody-based microbead cell separation (Miltenyi Biotec, Auburn, CA). The purity of the isolated cells is normally more than 90%.

### ChIP-seq

Briefly, cells were crosslinked with 1% formaldehyde for 10 min at room temperature. After quenching with 0.125M glycine for 5 min, cells were washed with PBS twice, and resuspended in Szak RIPA buffer and left on ice for at least 20 min before sonication. After sonication, immunoprecipitations were performed using 5 µg H3K4me3 (Abcam, ab8580) or RbIgG control antibody (Abcam, ab46540) from the chromatin fragments of 5×10^6^ cells. Deep sequencing libraries were constructed from 10 ng ChIP or Input DNA following Illumina protocol. 7 pM of final library product was sequenced either on GA_IIX_ or HiSeq2000.

### Genomic Sequencing Validation

PCR primers were designed for selected regions that contained novel SNVs and SHMs predicted by SHMseeqer in OCI-Ly1 cells (Supporting Information). PCR was performed using high fidelity Phusion polymerase (Thermo Scientific F-530L, Rockford, IL) on genomic DNA extracted from OCI-Ly1 cells. PCR products were purified using Qiagen MinElute PCR Purification Kit and sequenced directly by conventional Sanger sequencing method. Sequencing trace files were compared to reference genome to identify differences.

### Reporter Assay

*BCL6* and *BACH2* promoter sequences were amplified from BAC clones and cloned into pGL3 Luciferase Reporter basic vector (E1751, Promega) by using Cold Fusion cloning kit (Supporting Table S4) (SBI System Biosciences). Vectors that had correct sequences were subjected to QuickChange site-mutagenesis (Agilent, 200521) at positions where putative SHMs were identified by SHMseeqer (Supporting Table S4). After sequence validation, 1 ug vectors were transfected into 0.75×10^6^ OCI-Ly1 cells along with 0.1 ug control Renilla vectors by using Amaxa Nucleofactor kit SF (Lonza, VHCA-1002). Luciferase activity was measured using Dual Luciferase Reporter Assay system (E1910, Promega) and normalized to the Renilla activity.

### Gene Expression

RNA was isolated by TRIzol (15596-026, Life Technologies) and cDNA was made using Verso cDNA kit (Thermo Scientific, AB1453A). Quantitative real-time PCR was performed using a HT7900 Fast Real-Time PCR system (Applied Biosystems). Primer information can be found in Supporting Table S4. The expression level of the gene of interest is normalized relative to the expression level of house-keeping gene *HPRT*.

## Supporting Information

Figure S1 **SNV frequency of SHM hotspots in NB, GCB, OCI-Ly1 and OCI-Ly8.**
(DOC)

Figure S2 ***AICDA*** **expression in NB, GCB, OCI-Ly1, and OCI-Ly8.**
(DOC)

Figure S3 ***BCL6*** **and** ***BACH2*** **expression in NB, GCB, and OCI-Ly1.**
(DOC)

Table S1 **Lists of genes that have novel 5′ gene regulatory region SNVs in NB, GCB, OCI-Ly1, and OCI-Ly8, and genomic locations of novel SNVs identified in SHM hotspots.**
(XLS)

Table S2 **Stats of SHM hotspots identified using H3K4me3 ChIP-seq data in NB, GCB, OCI-Ly1, and OCI-Ly8.**
(XLS)

Table S3 **Stats of SHM hotspots in OCI-Ly1 identified using H3K4me2 ChIP-seq data.**
(XLS)

Table S4 **Primer sequences and reporter sequences used in the Sanger sequencing validation, gene expression study, and dual-luciferase assay.**
(XLS)
