# Supplementary material for: Genome-Wide Detection of Genes Targeted by Non-Ig Somatic Hypermutation in Lymphoma
Source: PLoS One. 2012 Jul 12;7(7):e40332. doi: 10.1371/journal.pone.0040332 (PMC3395700; doi:10.1371/journal.pone.0040332)
Supplement: Figure S1 — SNV frequency of SHM hotspots in NB, GCB, OCI-Ly1 and OCI-Ly8. (DOC) [file pone.0040332.s001.doc]

Supplementary Figure S1


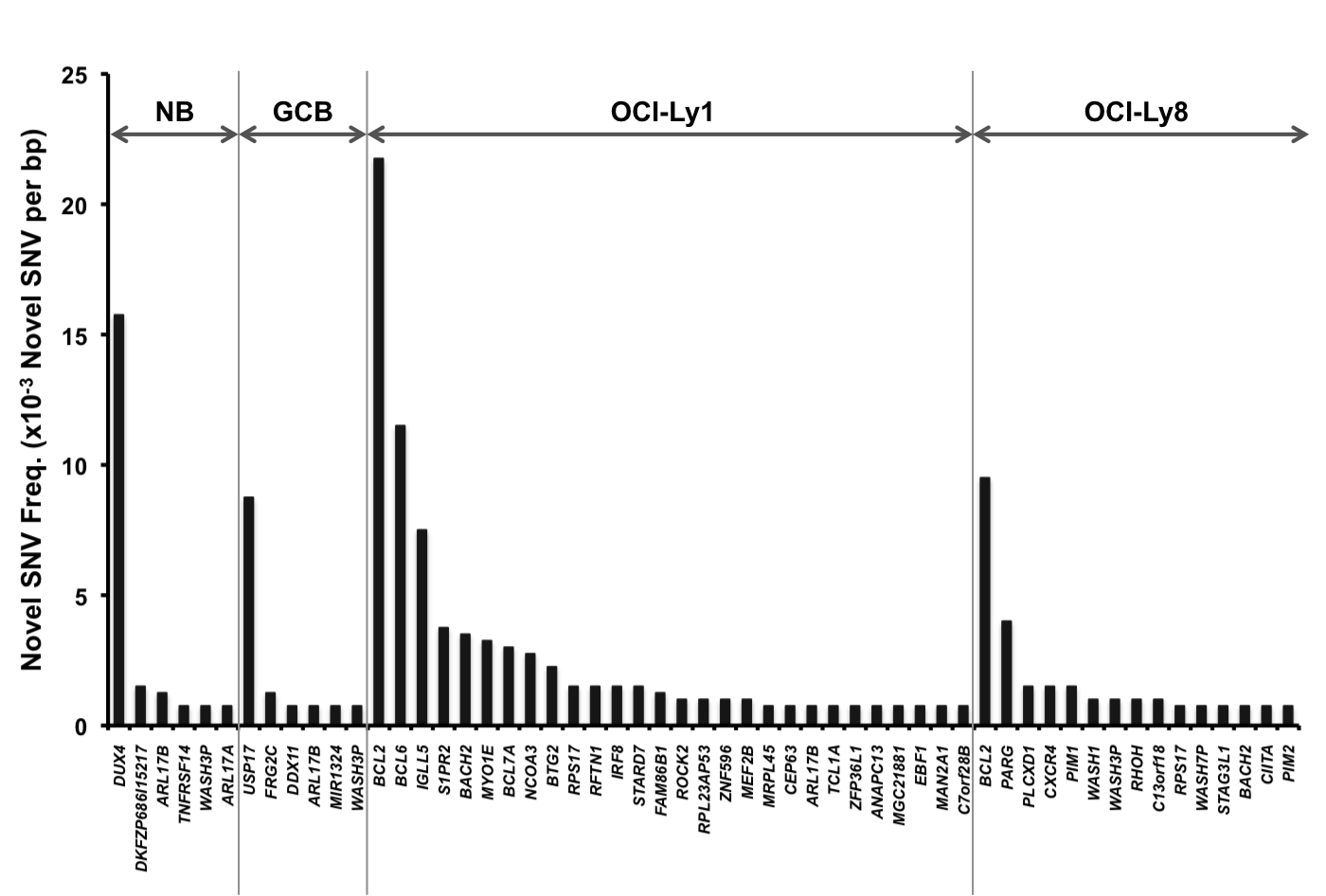


Supplementary Figure S1, Novel SNV frequency at SHM hotspot gene 5’ regulatory regions (TSS+⁄−2kb).
