# Supplementary figures and images for: Genome-Wide Detection of Genes Targeted by Non-Ig Somatic Hypermutation in Lymphoma
Source: PLoS One. 2012 Jul 12;7(7):e40332. doi: 10.1371/journal.pone.0040332 (PMC3395700; doi:10.1371/journal.pone.0040332)

Supplementary Figure S2


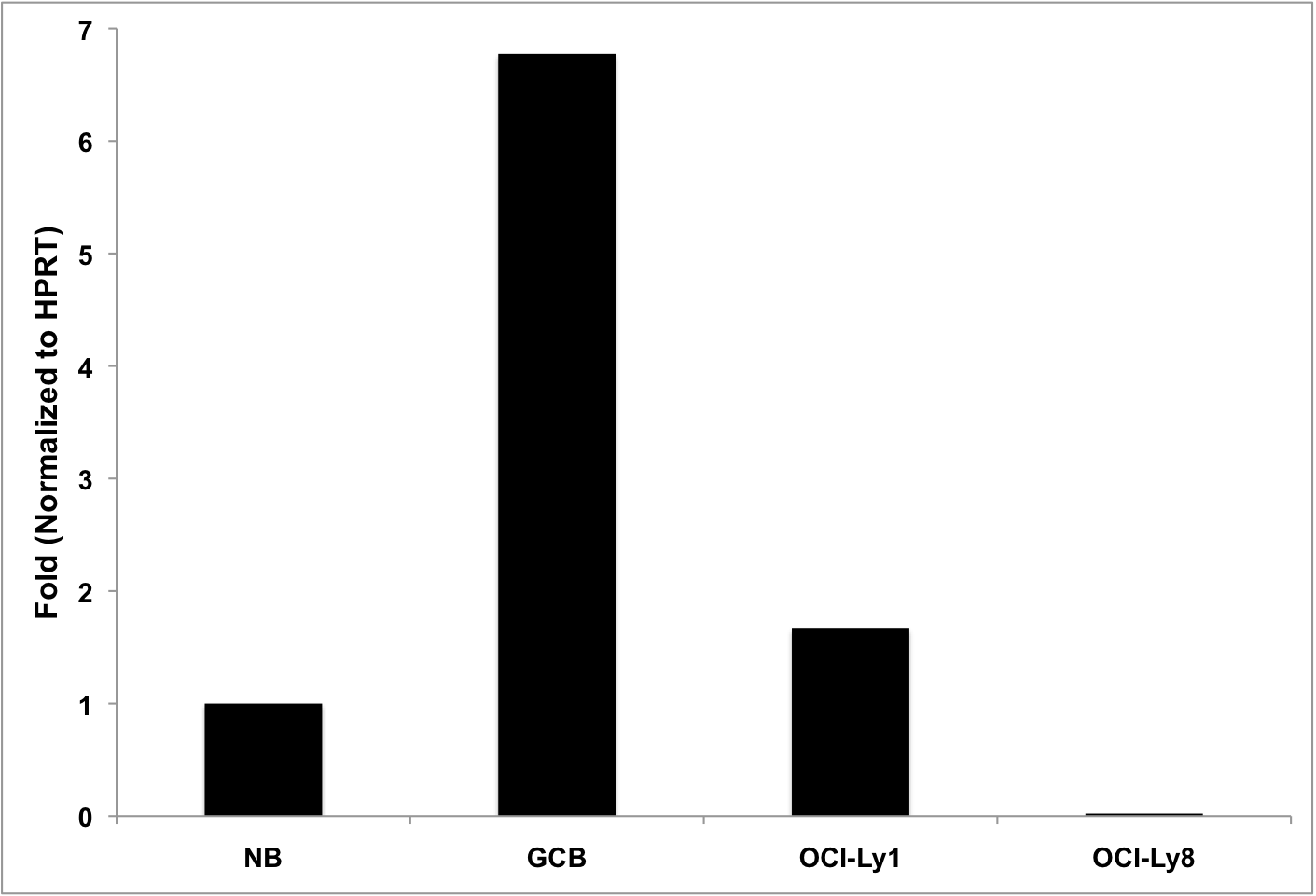


Supplementary Figure S2. *AICDA* Expression.

Supplement: Figure S2 — AICDA expression in NB, GCB, OCI-Ly1, and OCI-Ly8. (DOC) [file pone.0040332.s002.doc]
