# Supplementary material for: Genome-Wide Detection of Genes Targeted by Non-Ig Somatic Hypermutation in Lymphoma
Source: PLoS One. 2012 Jul 12;7(7):e40332. doi: 10.1371/journal.pone.0040332 (PMC3395700; doi:10.1371/journal.pone.0040332)
Supplement: Figure S3 — BCL6 and BACH2 expression in NB, GCB, and OCI-Ly1. (DOC) [file pone.0040332.s003.doc]

Supplementary Figure S3


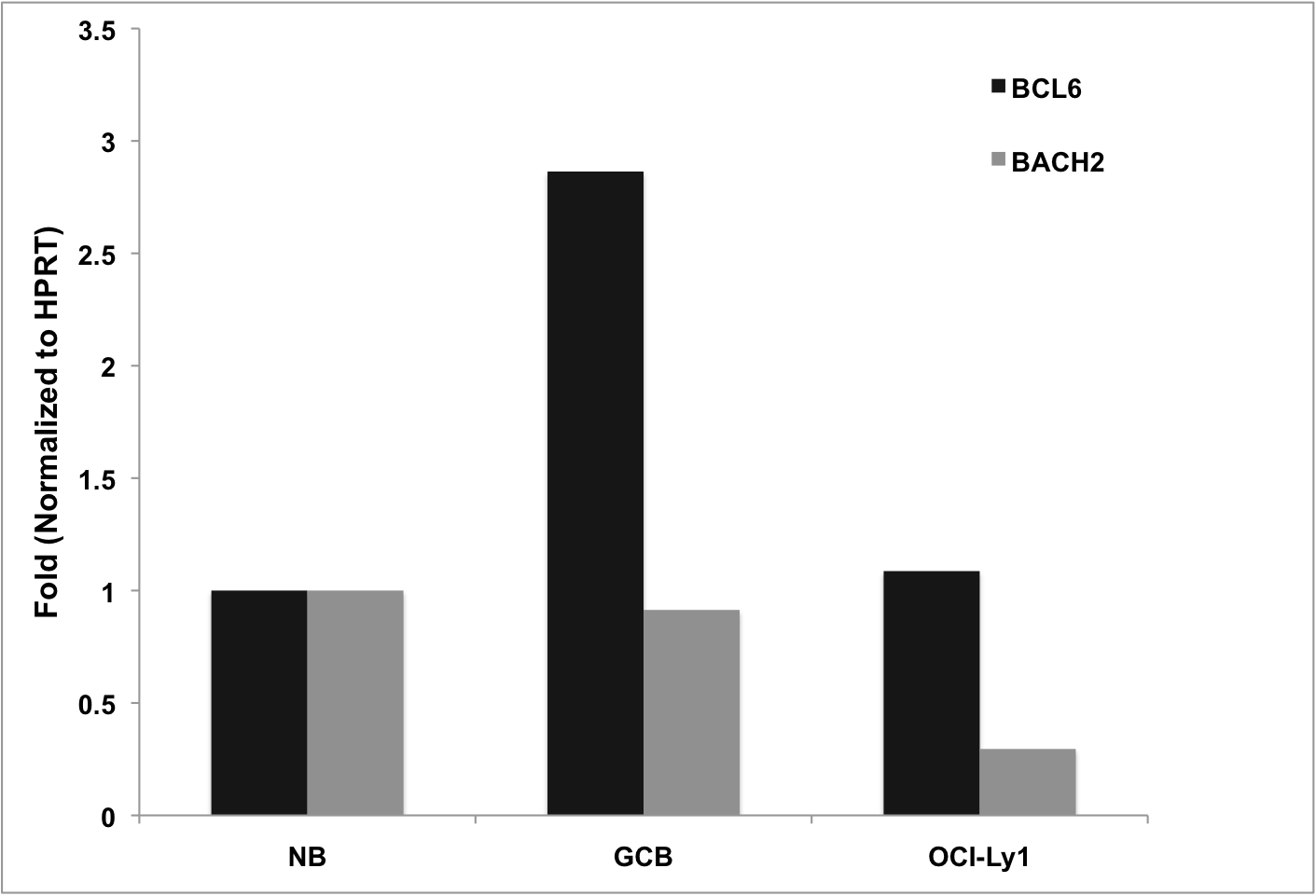


Supplementary Figure S3. *BCL6* and *BACH2* expression in NB, GCB, and OCI-Ly1 cells.
